# Intraperitoneal Dexamethasone As A New Method for Relieving Postoperative Shoulder Pain after Gynecologic Laparoscopy

**Published:** 2012-06-19

**Authors:** Zahra Asgari, Sima Mozafar-Jalali, Nasrin Faridi-tazehkand, Somayeh Sabet

**Affiliations:** 1 Department of Obstetrics and Gynecology, Arash Hospital, Tehran University of Medical Sciences, Tehran, Iran; 2 Department of Anesthesiology, Arash Hospital, Tehran University of Medical Sciences, Tehran, Iran

**Keywords:** Postoperative Pain, Intraperitoneal, Dexamethasone, Gynecologic, Laparoscopy

## Abstract

**Background:**

In this study, we tried to show the efficacy of Intraperitoneal dexamethasone on relieving shoulder pain after gynecologic laparoscopy.

**Materials and Methods:**

In this double-blind randomized clinical trial, 63 patients who were candidates for gynecologic laparoscopy were included. At the end of the procedure patients randomly received 16 mg dexamethasone (n=31) or placebo (n=32) intraperitoneally. Visual analogue scale (VAS) was used for clinical evaluation of pain severity during 24 hours after laparoscopy . A physician, who was not aware whether patients were treated with drug or placebo, evaluated the patients.

**Results:**

The severity of pain in the dexamethasone group within 0, 2, 4, 8, 12, 24 hours after procedure was significantly less than in the placebo group (p<0.001). The average consumption of opioids as analgesic/ sedative in the placebo group was more than the dexamethasone group (p=0.025).

**Conclusion:**

Findings of this study show that the prescription of 16 mg of dexametha- sone (single dose) in the peritoneal cavity may significantly reduce the severity of pain after Laparoscopy in comparison with placebo and may decrease the need for narcotics as pain relief (Registration Number: IRCT201105306640N1).

## Introduction

Today, laparoscopy is one of the most common methods of diagnosis and treatment used all over the world. It has various indications in abdominal and pelvic surgeries. Laparoscopic surgery has many advantages: patients recover very quickly thus requiring a shorter stay in hospital (1-3). Moreover, laparoscopy has less postoperative pain and the need for analgesics is less than open surgery (4)However, some degree of pain specially acute pain is probable after Laparoscopy. In such cases, opioid therapy is recommended (5).

Interestingly, post-laparoscopic pain is very different from that of post-laparatomy. In fact, the pain after laparotomy is somatic pain; whereas, patients experience visceral pain after laparoscopy (5). Shoulder pain is due to the carbon dioxide pneumoperitoneum that is created by the instigation of diaphragm during laparoscopy (6). This pain can be significant and various treatments are recommended for its relief. Several investigations have been conducted in order to find the cause of this pain. According to most of these investigations, the pain is attributed to peritoneal inflammation due to carbon dioxide pneumoperitoneum. Therefore, patients are recommended to take non steroidal anti inflammatory drugs (NSAIDs) (7). However, the use of NSAIDs for relieving postoperative pain is strongly controversial, because pneumoperitoneum is due to carbon dioxide which can cause some pathophysiologic changes in the bloodstream of kidneys that can interfere with the effects of NSAIDs.

Recently, the injection of local analgesics at the time of laparoscopic surgery, in order to relieve postoperative pain has become more common. One of such methods is the intra abdominal injection of analgesics such as lidocaine and bupivacaine (8-13). However, there are no definitive evidence about effectiveness of these methods and they are controversial. As mentioned above, the cause of postoperative pain is inflammation of peritoneum due to carbon dioxide pneumoperitoneum. Therefore, it seems the pain can be reduced by the reduction of inflammation.

Since steroids have been used for reducing inflammation, they may be considered as alternatives for relieving pain. Dexamethasone is a strong long acting Glucocorticoid and it is widely used after surgery (14, 15). It has been demonstrated that injecting a single dose of dexamethasone may prevent post operative nausea (16).

Recently, it has been established that higher doses of dexamethasone and other glucocorticoids are effective in relieving postoperative pain in foot surgeries, breast surgeries, laparoscopic cholecystectomy and spinal column surgeries (17-24).

Although, the effects of dexamethasone have not yet been established, it is probable that pain relief is caused by the inhibition of prostaglandins production . There is an other theory which states that dexamethasone acts by reducing the amount of 5-Hydroxytryptamine (5HT) which is returned into the central nervous system or by increasing the permeability of the blood-brain barrier for serum proteins. So far, according to available information, there is not a published study with high quality methodology about effectiveness of intraperitoneal dexamethasone. However, intravenous dexamethasone has been previously used for relieving nausea after laparoscopy (25-27).

In this study, we compare the effectiveness of intraperitoneal dexamethasone with placebo in patients which have undergone gynecologic laparoscopy in a double-blind randomized clinical trial.

## Materials and Methods

This study is a double blind randomized clinical trial that has been approved by the Ethical Committee of Tehran University of Medical Science. We enrolled women who had undergone gynecologic laparoscopy in Arash Hospital during 2009-2010. According to the inclusion criteria for participation in this study, only women between 18-70 years old with gynecologic indication for laparoscopy were included. Furthermore, since the general condition of patients was important and in order to allow the questions of investigators to be answered, the patients were allocated to groups I or II according to the American Society of Anesthesiologists (ASA) physical classification system. Patients with a history of diabetes mellitus, abdominal surgery, drug reaction to dexamethasone and those previously treated with steroids were excluded from the study. All the patients signed a written consent form after being made aware of the full procedure before participating in this study. A gynecologist gathered the basic information needed for this study such as age, fertility condition (gravidity, parity), weight, height, body mass index (BMI) and the type of laparoscopic surgery they had undergone from each patient before assigning them to one of the two groups.

The patients were then divided to two groups, and those in the first group received intraperitoneal dexamethasone while those in the second group received placebo. Block randomization method with four blocks was used to assign the patients into groups. The treatment option for the groups was selected by chance by drawing lots. At the end of selection, the number of people who had been selected in each block were equal. All patients underwent anesthesia. At the end of the surgery, the surgeon injected 16 mg of dexamethasone into the peritoneum of patients from the first group and 16 cc of normal saline as placebo to patients in the second group.

Patients were asked about severity of shoulder pain within 2, 4, 8, 12, 24 hours after laparoscopy. Severity of pain was assessed by visual analogous scale (VAS) method. In this method, the intensity of pain was classified from zero to 10. Painlessness was scored as 0 and the maximum severity of pain that patients experienced was scored as 10.

According to VAS method, patients reported their severity of pain within mentioned periods. 25 mg of pethidine that was diluted in 10 cc normal saline was injected intravenously. This dose was repeated every 4 hours according to patients' demand. The total amount of pethidine injected within 24 hours after surgery was recorded for each patient.

Collected data were analyzed by SPSS 13. Quantitative data were shown as mean and standard deviation and qualitative data were shown as frequency. Chi-square was used to compare the two groups of qualitative data and the ttest was used to compare quantitative data in two groups if our distribution was normal. When our distribution was not normal, nonparametric tests were used. P-value less than 0.05 was considered as significant.

## Results

In this study, 63 patients in two groups were evaluated. The first group included patients who received intraperitoneal dexamethasone (31 patients) and the second group included patients who received placebo (32 patients). Patients were aged between 18-47 and according to our findings, there was no significant difference between the two groups regarding age, BMI and the physical condition based on ASA. According to the results there is no statistically significant difference between the two groups in the mean time of Laparoscopic surgery which is 51.1 ± 35.8 minutes in the first group (dexamethasone) and 53.7 ± 35.6 minutes in the second group.

As mentioned above, the patients' pain severity increased in the placebo group and after 8 hours it started to decrease; whereas, in the dexamethasone group, the average pain severity was less than 3. In the dexamethasone group, during recovery and 2, 4, 8, 12, 24 hours after surgery, the pain severity was significantly less than in the placebo group. This difference is less than other stages of the study after recovery. Also, passing time and other general factors such as age, BMI and long-time laparoscopic surgery may influence the patient’s pain severity; so we used analysis of the variance of repeated measurements to try and determine whether these factors influence pain severity.

The results of this study show that reduction of pain severity in dexamethasone group is independent from other factors and dexamethasone can relieve the pain severity by itself (p<0.001). The first time of prescribing opioid as an analgesic in dexamethasone group and placebo group was after 1 hour. Generally, 83% of patients treated with dexamethasone and 78% of patients treated with placebo requested an analgesic during one hour after surgery. There are no statistically significant differences regarding the first time of demanding analgesics.

The average amount of opioid (pethidine) administered was 27.5 ± 7.6 mg in the dexamethasone group and 35.9 ± 18.9 mg in the placebo group. The prescribed amount of opioid as an analgesic in the placebo group was significantly more than that used in the dexamethasone group (p=0.025). We found no complications such as wound infection and delay in wound healing during the period of investigation.

## Discussion

The main goal of designing this study is to answer the question of whether a single dose of dexamethasone injected into the peritoneal cavity can relieve the pain severity after laparoscopic surgery or not? We believe this to be a novel investigation seeing as no similar studies have previously been conducted. The results of this study, show that injection of dexamethasone in the peritoneum after laparoscopic surgery can significantly relieve postoperative pain. Also, the patients treated with dexamethasone requested fewer narcotics compared to the patients treated with the placebo.

Prevention and relief of postoperative pain and its complications such as nausea and vomiting are important concerns in the care of patients after laparoscopy. In addition, this plays an important role in the improvement of patients´ general condition (28). For controlling postoperative pain, non-opioid analgesics are usually considered. However, because of the known complications of non-opioid analgesics, we tried to find new drugs to relieve postoperative pain. In fact, the main key of this investigation has been based on the control and treatment of postoperative pain after laparoscopy (29).

As previously mentioned, the mechanism of pain after laparascopy is completely different from the pain after Laparatomy. Pain after Laparotomy is a somatic pain but Laparoscopy is followed by a visceral pain (7, 30). Some investigations into the cause of this pain have attributed it to the inflammation of the peritoneum that has been created by carbon dioxide pneumoperitoneum. In such cases NSAIDs have been recommended for relieving pain (6, 7).

In the recent years local analgesics such as lidocaine and bupivacaine have been commonly used to relieve postoperative pain (25, 26). Intraperitonealy administered dexamethasone was used in this study. Although application of these drugs are controversial, but most experts believe that the inflammatory process in the peritoneal cavity is the best reason for shoulder pain after laparoscopic surgery. Thus, non-inflammatory agents such as corticosteroids are able to relieve shoulder pain severity via reduction of inflammation after laparoscopy.

Dexamethasone as a corticosteroid has been widely used for the treatment of some complications such as nausea and vomiting and has been effective.

Although the analgesic effects of corticosteroids have been shown in the clinical settings, it has not yet been determined whether it can be effective in the reduction of postoperative pain or not?

## Conclusion

In this study, we injected dexamethasone directly to the inflamed location and we succeeded to show that dexamethasone can relieve postoperative pain and reduce the need for opioids. Nevertheless, the question still remains whether or not the systemic (intravenous) dexamethasone can cause such effects?

Although applied amounts of dexamethasone has no serious side effects and it has been widely used, if higher doses are administered some side effects may be experienced such as gastrointestinal bleeding, increasing infection rate and delayed wound healing.

In a meta-analysis of 52 investigations 15-30 mg/kg of methylprendnisolone was prescribed for 1900 patients. No significant difference was reported regarding serious side effects in comparison with the control group (31). Some of these studies had been conducted in the critical wards (such as heart surgery, neurosurgery and trauma ward) rather than in elective and laparoscopic surgery wards. In fact, the only significant effect on patients was reduction of pulmonary complications (this complication is more common in patients who have a fracture). Review literatures could not find any clinical study that show the use of a single dose of corticosteroids can cause serious side-effects for the patients. Results of our study show that 16 mg dexamethasone can significantly relieve the pain severity after gynecologic laparoscopy in comparison with placebo; and also these patients need less opioid. This method, which has no serious side effects, can be used as a preventive method to control postoperative pain after laparoscopic treatment.
